# Ghrelin, leptin, adiponectin, and resistin levels in sleep apnea syndrome: Role of obesity

**DOI:** 10.4103/1817-1737.65050

**Published:** 2010

**Authors:** Ahmet Ursavas, Yesim Ozarda Ilcol, Nazan Nalci, Mehmet Karadag, Ercument Ege

**Affiliations:** *Department of Pulmonary Medicine, School of Medicine, University of Uludag, Bursa, Turkey*; 1 *Department of Biochemistry, School of Medicine, University of Uludag, Bursa, Turkey*

**Keywords:** Adipokines, obesity, sleep apnea

## Abstract

**AIM:**

The aim of this study was to investigate the relationship among plasma leptin, ghrelin, adiponectin, resistin levels, and obstructive sleep apnea syndrome (OSAS).

**METHODS:**

Fifty-five consecutive newly diagnosed OSAS patients and 15 age-matched nonapneic controls were enrolled in this study. After sleep study between 8:00 AM and 9:00 AM on the morning, venous blood was obtained in the fasting state to measure ghrelin and adipokines.

**RESULTS:**

Serum ghrelin levels of OSAS group were significantly (*P* < 0.05) higher than those of the control group. No significant difference was noted in the levels of leptin, adiponectin, and resistin in OSAS group when compared to controls. There was a significant positive correlation between ghrelin and apnea–hypopnea index (AHI) (r = 0.237, *P* < 0.05) or the Epworth sleepiness scale (ESS) (r = 0.28, *P* < 0.05). There was also a significant positive correlation between leptin and body mass index (r = 0.592, *P* < 0.0001). No significant correlation was observed between leptin, adiponectin, resistin, and any polysomnographic parameters.

**CONCLUSION:**

Our findings demonstrated that serum ghrelin levels were higher in OSAS patients than those of control group and correlated with AHI and ESS. Further studies are needed to clarify the complex relation among OSAS, obesity, adipokines, and ghrelin.

Obstructive sleep apnea syndrome (OSAS) is characterized by repetitive collapse of the upper airway during sleep, resulting in impedance to airflow and oxygen desaturations, which cause arousal from sleep.[1] OSAS is strongly associated with obesity, approximately 70% of OSAS patients are obese and obesity is the only reversible risk factor of importance.[2,3]

Leptin is a circulating hormone that is expressed abundantly throughout the body specifically in adipose tissue. The primary role of leptin is in controlling appetite.[4] Adiponectin is the adipokine that circulates at the highest. Resistin is a member of cysteine-rich secretory proteins.[5] Leptin and resistin have been shown to exert potent pro-inflammatory properties by upregulating pro-inflammatory cytokines. On the other hand, adiponectin reduces the production and activity inflammatory cytokine.[6] Ghrelin is a 28 amino acid peptide hormone, has an appetite-stimulating effect and controls the energy balance.[7]

OSAS is strongly associated with obesity and inflammation. With the growing prevalence of obesity, scientific interest in the biology of adipose tissue has been extended to the secretory products of adipocytes. Human obesity is associated with increased leptin, resistin levels, and decreased adiponectin levels.[8–11] The plasma ghrelin level is lower in obese subjects than in nonobese subjects.[7] Several studies suggest that there are significant relationships among obesity, adipokines, systemic inflammation, and atherosclerosis.[12,13]

Although association of obesity, leptin, ghrelin, and inflammations well documented, role of adipokines and ghrelin in OSAS remains controversial. The objectives of this study were twofold. First, we sought to determine and compare the levels of serum leptin, ghrelin, resistin, and adiponectin in OSAS patients and nonapneic controls. Second, we sought to determine relationship between adipokines each other and some sleep parameters. To the best of our knowledge, this is the first reported attempt to assess these adipokines and ghrelin in OSAS patients.

## Methods

### Subjects

A cross-sectional study was performed, which included subjects referred to the same sleep center for the investigation for snoring or possible OSAS. Fifty-five consecutive newly diagnosed OSAS patients and 15 age-matched nonapneic controls were enrolled in this study. Subjects with heart failure, chronic renal failure, chronic obstructive pulmonary disease, on systemic steroid treatments, and on hormonal replacement therapy were excluded from the study.

All subjects were asked to complete a questionnaire about the presence of any history of snoring, witnessed apnea, excessive daytime sleepiness, Epworth Sleepiness Scale (ESS), medical history, and medication. Demographic information (age, gender, and smoking habits) and anthropometric measurements including height, weight, body mass index (BMI): weight/height (kg/m^2^) were obtained on presentation to the sleep center.

The study protocol was approved by the Ethics Committee of the Uludağ University Medical Hospital, Bursa, Turkey. All patients gave written informed consent to participate in this study.

### Sleep analysis

Full polysomnography (PSG) monitoring was performed on all participants using the Compumedics P-series Sleep System (Compumedics Sleep: Melbourne, Australia). All participants reported to the sleep laboratory at approximately 8.30 PM, and PSG was initiated at approximately 8.30 PM. Polysomnographic recordings included two electro-encephalography (EEG) channels (C3/A2 and O2/A1), two electro-oculogram (EOG) channels, one submental electromyogram (EMG) channel, and one electrocardiography (ECG) channel. Ventilatory monitoring included recording of oronasal airflow (with an oronasal thermistor), hemoglobin oxygen saturation by pulse oximetry (SaO_2_ was measured via a finger oximeter), respiratory movement (with an inductive plethysmography), including chest and abdomen, and body position.

Sleep staging was performed according to the standard criteria of Rechtschaffen and Kales.[14] To assess ventilation during sleep, nasal airflow was analyzed carefully. Apnea was defined as episodes of airflow cessation lasting ≥10 s. Hypopnea was defined as episodes lasting ≥10 s, with reductions of thermistor signal amplitude ≥50% and an associated fall of ≥3% in oxygen saturation, or an arousal that lasted ≥10 s. The sum of time spent in apnea and hypopnea was divided by the total sleep time to determine the apnea–hypopnea index (AHI). Subjects with AHI ≥ 5 were considered to have OSAS. Subjects with AHI < 5 were included in the control group.

### Serum leptin, ghrelin, adiponectin, and resistin assays

After sleep study between 8:00 AM and 9:00 AM on the morning, venous blood was obtained in the fasting state to measure ghrelin and adipokines. Blood samples were centrifuged with in 30 min at 4°C for at 3000 × *g* for 10 min. Serum samples were stored at –80°C until performance of assay.

Serum adiponectin, leptin, and ghrelin levels were measured by radioimmunoassay using a commercially available kit (Linco Research, St. Charles, MO, USA). All serum samples from individual subjects were assayed in duplicate in the same test battery. Within and between assay coefficients of variations were <6% and <7%, <5% and <8%, or <7% and <10% for adiponectin, leptin, or ghrelin, respectively. The lowest level of human leptin and ghrelin that can be detected by the assay kits was 0.5 ng/mL and 95 pg/mL when using a 100 µL undiluted serum sample, respectively. For serum adiponectin measurement, serum samples were diluted by 1/500 with assay buffer as advised. The lowest level of human adiponectin that can be detected by the assay was 1 ng/mL when using a 100 µL of diluted (1/500) serum sample.

Serum resistin levels were measured using a commercially available ELISA kit (Linco Research, St. Charles, MO, USA). All serum samples from individual subjects were assayed in duplicate in the same test battery. Within and between assay coefficients of variations were <5% and <7%, respectively. The lowest level of human resistin that can be detected by this assay was 0.16 ng/mL.

### Statistical analysis

Statistical analysis was performed using the SPSS package for Windows, version 13.0 (SPSS, Inc., Chicago, IL, USA). Comparisons between data of the OSAS and control groups were carried out with Student’s *t*-test, Chi-square test, and the Mann–Whitney *U*-test. The concordance of normal distribution of all variables was calculated with the Shapiro–Wilk test before comparison between OSAS and control groups. If the data were not normally distributed, we used nonparametric tests for dependant variables. Relationship between serum adipokines levels and ESS, number of total apnea, number of total respiratory events, time spent in apnea and hypopnea, average oxygen desaturation, oxygen desaturation index, REM sleep, slow wave sleep, and respiratory arousal index were calculated using the Pearson correlation analysis. *P* value <0.05 was considered statistically significant.

## Results

### Baseline characteristics

Baseline characteristic of the study population are shown in Table 1. There were 10 diabetic and 15 hypertensive patients in OSAS group. There was no significant difference in age, gender, BMI, smoking habit, and snoring between the two groups. In the OSAS group, excessive daytime sleepiness and ESS scores were significantly higher than those of the control group (*P* < 0.001). There were no significant differences in the total sleeping time, sleep efficiency, and baseline oxygen saturation between the two groups. Significant differences (*P* < 0.05–0.001) in sleep Stages 3 and 4, arousal (per hour), AHI, duration of apnea–hypopnea, oxygen desaturation index, average oxygen saturation during sleep, average oxygen desaturation, and length of time spent with an oxygen saturation <90% were noted when the OSAS group was compared to control group [Table 1].

**Table 1:** Baseline clinical and polysomnographic characteristics of the study sample (mean ± SEM)

	OSAS	Control	*P* value
Age	51.1 ± 1.2	48.4 ± 3.0	NS
Body mass index, kg/m^2^	32.5 ± 0.9	31.6 ± 1.8	NS
Smoking habits (pack-years)	17.3 ± 1.9	19.5 ± 7.0	NS
Epworth sleepiness scale	11.2 ± 5.9	5.6 ± 0.7	<0.05
Total sleep time (TST), h	6.2 ± 0.9	6.5 ± 0.8	NS
Sleep efficiency, %	82.5 ± 1.3	73.3 ± 4.3	NS
Stages 3 and 4 (%TST)	8.5 ± 1.1	18.5 ± 2.4	<0.05
Rapid eye movement (%TST)	10.5 ± 1.7	19.2 ± 1.7	<0.05
AHI, per hour	43.5 ± 3.6	2.8 ± 0.4	<0.0001
Duration in apnea–hypopnea, min	124.4 ± 13.3	5.3 ± 1.1	<0.0001
Arousal, per hour	39.2 ± 2.7	14.7 ± 1.3	<0.0001
Baseline oxygen saturation, %	92.7 ± 0.5	93.6 ± 0.9	NS
Average oxygen saturation % in sleep	86.8 ± 1.1	92.2 ± 1.5	<0.05
Average desaturation, %	11.6 ± 1.2	3.9 ± 1.4	<0.0001
Oxygen saturation <90%, length of time, min	98.6 ± 16.4	37.8 ± 17.4	<0.05

NS: Statistically insignificant

### Serum leptin, grelin, adiponectin, and resistin levels

Serum levels of leptin, ghrelin, adiponectin, and resistin of the two groups are shown in Table 2. The serum levels of ghrelin in the OSAS group (564 ± 44 pg/mL) were significantly (*P* < 0.05) higher than those of the control group (403 ± 90 pg/mL). No significant difference was noted in the levels of leptin, adiponectin, and resistin in OSAS group when compared to controls.

**Figure 1:**
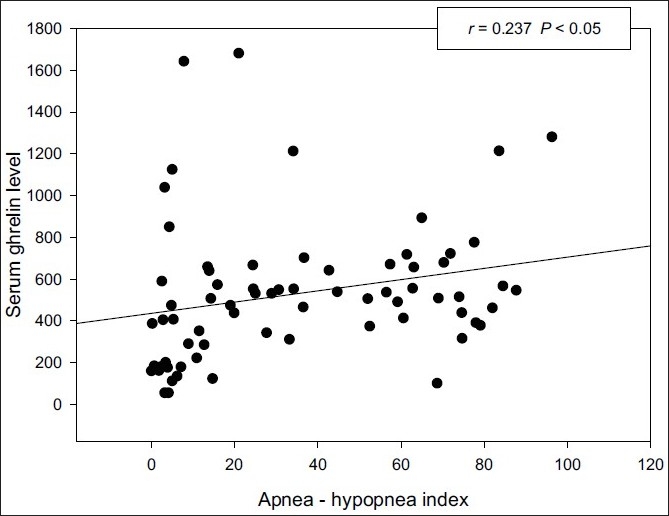
Correlation between serum ghrelin levels and apnea–hypopnea index

**Figure 2:**
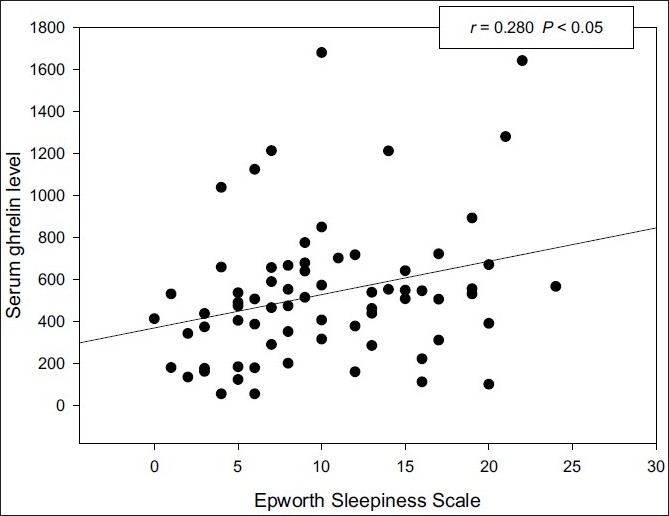
Correlation between serum ghrelin levels and Epworth sleepiness scale

**Figure 3:**
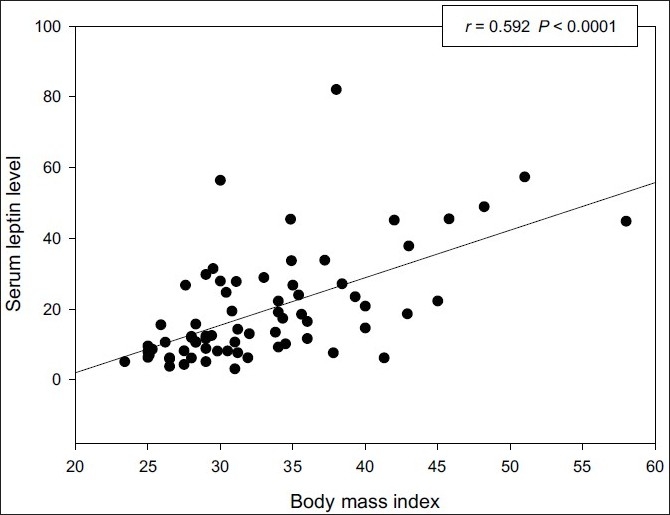
Correlation between serum leptin levels and BMI

There was a significant positive correlation between ghrelin and AHI [Figure 1] or ESS (*r* = 0.28, *P* < 0.05) [Figure 2], and negative correlation was also noted between ghrelin and resistin (*r* = –0.39, *P* < 0.05). There was a significant positive correlation between leptin and BMI (*r* = 0.59, *P* < 0.0001) [Figure 3]. There was no significant correlation between adipokines and any polysomnographic parameters.

**Table 2:** The plasma levels of leptin, ghrelin, adiponectin, and resistin (mean ± SEM)

	OSAS	Control	*P* value
Leptin, ng/mL	10.9 ± 2.7	9.4 ± 0.9	NS
Ghrelin, pg/mL	564 ± 44	403 ± 90	<0.05
Adiponectin, □g/mL	7.7 ± 0.7	9.1 ± 1.7	NS
Resistin, ng/mL	3.5 ± 0.3	3.1 ± 0.3	NS

NS: Statistically insignificant

## Discussion

The results of our study indicated that the serum levels of ghrelin in the OSAS group were significantly higher than those of the nonapneic control group. There was a significant positive correlation among serum levels of ghrelin, AHI, and ESS. No significant difference was noted in the levels of leptin, adiponectin, and resistin in OSAS group compared to controls. There was a significant positive correlation between leptin and BMI.

Serum level of leptin is correlated with BMI in humans.[15] Leptin levels were found increased in patients with OSAS. However, several studies suggested that these abnormal leptin levels may be related to the obesity, sympathetic activity, and hypoxia in patients with OSAS.[16–18] Tatsumi *et al*.[17] indicated that circulating leptin levels were correlated with body mass index (BMI), visceral fat accumulation (VFA), subcutaneous fat accumulation (SFA), apnea–hypopnea index (AHI), sleep mean arterial oxygen saturation, and sleep lowest arterial oxygen saturation. Multiple regression analysis revealed that average oxygen saturation and lowest oxygen saturation were explanatory variables for serum leptin values, but AHI (*P* = 0.054), BMI, VFA, and SFA were not. It was suggested that sleep hypoxemia may be the main determinant of circulating leptin levels. Shimura *et al*.[18] demonstrated that circulating leptin levels correlated with BMI, VFA, and SFA, but not with PaO_2_ or sleep mean arterial oxygen saturation. They reported that leptin levels were higher in the hypercapnic group than in the eucapnic group. Serum leptin was the only predictor for the presence of hypercapnia in logistic regression analysis. Shimizu *et al*.[19] reported that plasma leptin reached a peak level at 00:00 hours in patients with OSAS, both with and without continuous positive airway pressure (CPAP) treatment. The magnitude of the decrease in leptin levels after CPAP treatment was significantly correlated with cardiac sympathetic function measured before CPAP treatment. They suggested that serum leptin levels in the morning may be a marker of improved cardiac sympathetic nerve function.

On the other hand, Barcelo *et al*.[20] compared the levels of leptin in four groups, obese and nonobese with OSAS compared with obese and nonobese controls. They did not find any significant difference in the leptin levels between the groups. In recent study, Sharma *et al*.[21] reported that no significant difference was noted in levels of fasting blood sugar, insulin resistance, leptin, or adiponectin in OSAS group compared the obese controls. In this study, we showed that no significant difference was noted in the levels of leptin in OSAS group when compared to controls, there was not significant correlation among AHI, mean oxygen saturation in sleep, mean oxygen desaturation in sleep, oxygen desaturation index, and serum leptin levels. We only found a significant positive correlation between leptin levels and BMI.

Adiponectin has the biological effects of anti-inflammatory, anti-atherosclerosis, increasing insulin sensitivity and decreasing insulin resistance.[22] It has been reported that there is a significant relationship between OSAS and decreased serum level of adiponectin;[23] however, other studies found a normal or increased adiponectin level in patients with OSAS.[21,24,25] Zhang *et al*.[23] reported that serum adiponectin levels were significantly lower in the OSAS group than in the control. Makino *et al*.[24] examined that 213 patients with OSAS were divided into three groups: 30 with mild, 98 moderate, and 85 with severe OSAS. They determined plasma adiponectin levels were not different among mild, moderate, and severe OSAS groups. They suggested that plasma adiponectin was more closely related to obesity than to sleep apnea. Tauman *et al*.[25] indicated that adiponectin levels were lower in obese than nonobese children and were inversely correlated with BMI Z scores but not with AHI. In this study, we showed that no significant difference was noted in the levels of adiponectin in OSAS group compared to controls.

Resistin is new white adipose tissue hormone. Its linkage to obesity and OSAS were controversial in previous studies.[25–28] Harsch *et al*.[27] reported that while resistin levels were positively correlated with IL-6, CRP, ICAM-1, and leptin, negatively correlated with insulin sensitivity index. No correlation was observed between BMI or AHI and resistin levels. They found that resistin remained unchanged during CPAP therapy. In the recent study, adiponectin and resistin levels were compared with in simple obesity, obese OSAS patients, and multiple symmetrical lipomatosis (LBS), and they found no significant differences in serum adiponectin and resistin levels between these groups.[28] In this study, we showed that no significant difference was noted in the levels of resistin in OSAS group compared to controls.

Serum ghrelin levels are inversely related to changes of body weight. It is highest in anorectic subjects and low in obese persons.[29] Relationship between serum ghrelin level and OSAS is controversial. It has been suggested that reduced total sleep time can lead to alterations in parameters of glucose tolerance and dysregulation of appetite.[30] OSAS also involves respiratory stress in addition to sleep loss and sleep fragmentation, and hypoxia and hypercapnia also result in increases in sympathetic nerve activity. Ghrelin release may be related to sympathetic activation in OSAS. Harsch *et al*.[31] showed that baseline plasma ghrelin levels were significantly higher in OSAS patients than in controls. After 2 days of CPAP treatment, plasma ghrelin decreased in almost all OSAS patients to levels that were only slightly higher than those of controls. Ulukavak *et al*.[32] investigated serum leptin and ghrelin levels in obese patients with OSAS in comparison with equally obese controls without OSAS. They indicated that significantly higher serum leptin levels were found in OSAS patients compared to controls, but there was no significant difference in serum ghrelin levels between OSAS patients and controls. In this study, we showed that the circulating levels of ghrelin in the OSAS group were significantly higher than those of the control group and there was a significant positive correlation between ghrelin and AHI.

Potential limitations of this study merit consideration. First, we used oronasal thermistor for measured oronasal air flow. Second, this is a cross-sectional study and our sample size is limited. Finally, our control group subjects were not healthy people from population.

In conclusion, we found no relationship between adipokines and OSAS. On the contrary, serum levels of ghrelin was higher in OSAS patients. We suggest that there are several confounding factors such as obesity, repetitive hypoxia, hypercapnia, and sympathetic activation which can be influence serum adipokines and ghrelin levels in patients with OSAS. These factors may be reason of inconsistent results in several studies.
